# Post‐translational modifications in diabetic cardiomyopathy

**DOI:** 10.1111/jcmm.18158

**Published:** 2024-03-17

**Authors:** Zhi Li, Jie Chen, Hailong Huang, Qianru Zhan, Fengzhi Wang, Zihan Chen, Xinwei Lu, Guozhe Sun

**Affiliations:** ^1^ Department of Cardiology The First Hospital of China Medical University Shenyang China; ^2^ Department of Obstetrics and Gynecology Shengjing Hospital of China Medical University Shenyang China; ^3^ Department of Neurology, People's Hospital of Liaoning Province People's Hospital of China Medical University Shenyang China; ^4^ Department of Cardiology Siping Central People's Hospital Siping China

**Keywords:** acetylation, diabetic cardiomyopathy, methylation, O‐GlcNAcylation, phosphorylation, post‐translational modifications, ubiquitination

## Abstract

The increasing attention towards diabetic cardiomyopathy as a distinctive complication of diabetes mellitus has highlighted the need for standardized diagnostic criteria and targeted treatment approaches in clinical practice. Ongoing research is gradually unravelling the pathogenesis of diabetic cardiomyopathy, with a particular emphasis on investigating various post‐translational modifications. These modifications dynamically regulate protein function in response to changes in the internal and external environment, and their disturbance of homeostasis holds significant relevance for the development of chronic ailments. This review provides a comprehensive overview of the common post‐translational modifications involved in the initiation and progression of diabetic cardiomyopathy, including O‐GlcNAcylation, phosphorylation, methylation, acetylation and ubiquitination. Additionally, the review discusses drug development strategies for targeting key post‐translational modification targets, such as agonists, inhibitors and PROTAC (proteolysis targeting chimaera) technology that targets E3 ubiquitin ligases.

## 1 INTRODUCTION

Post‐translational modifications (PTMs) enhance the functional diversity of the proteome by introducing covalent additions of functional groups or proteins, proteolytic cleavage of regulatory subunits or protein degradation.^1^ The utilization of mass spectrometry‐based proteomics, particularly electrospray ionization mass spectrometry, has progressively unveiled the magnitude and prevalence of PTMs, highlighting its complexity.^2^ To date, over 200 PTMs have been identified. These chemical modifications, such as phosphorylation, glycosylation, ubiquitination, nitrosylation, methylation, acetylation, lipidation and protein hydrolysis, play a crucial role in cellular and subcellular compartments. These modifications can be reversible, depending on their nature.^3^ They dynamically modulate homeostasis in response to environmental changes and regulate multiple cellular signals. Consequently, studying these PTMs is of great significance, particularly in the context of chronic diseases such as cardiovascular disease, cancer, neurodegenerative diseases and diabetes.

**FIGURE 1:**
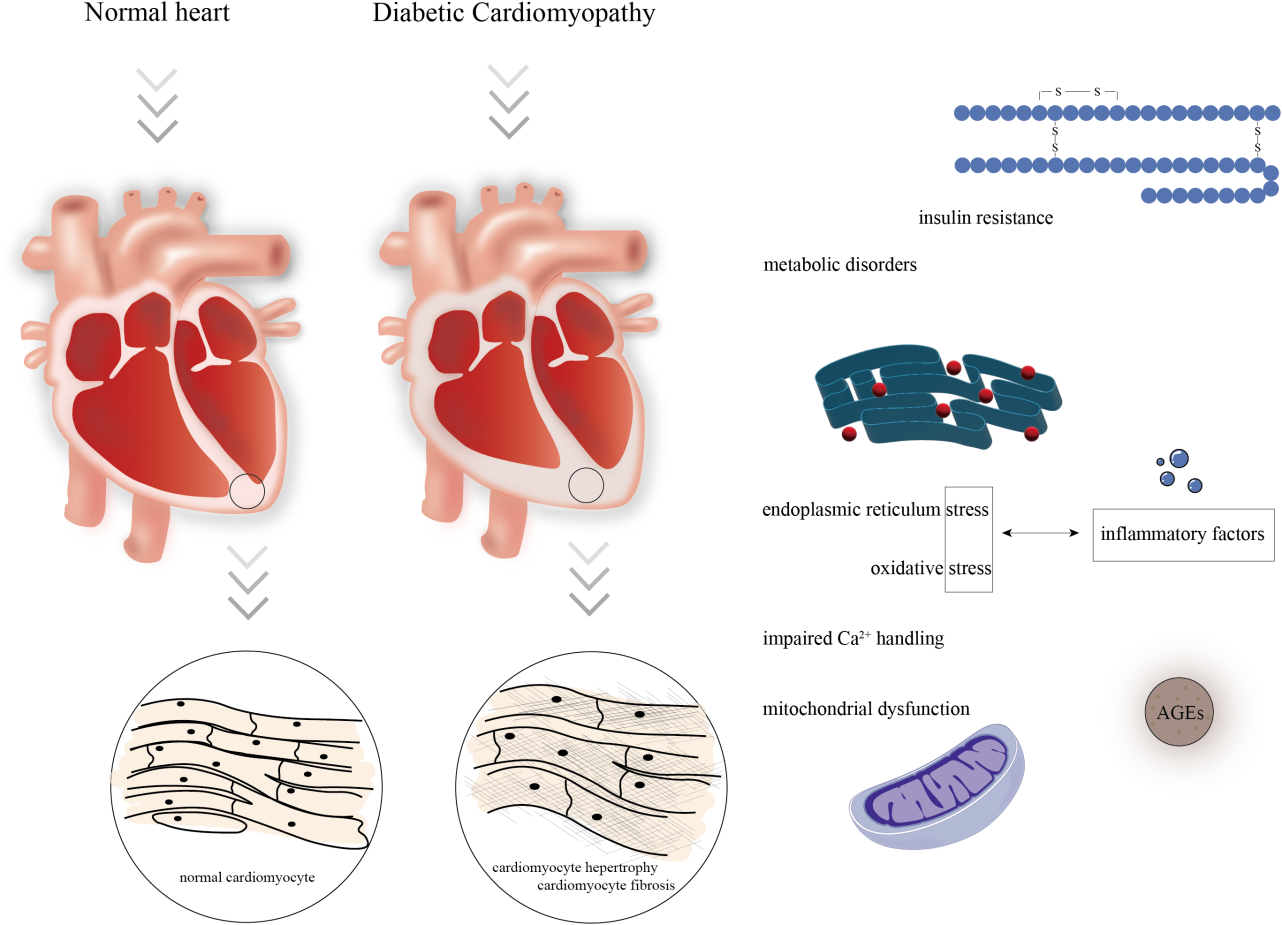
Pathogenesis associated with diabetic cardiomyopathy. An intricate interplay among systemic insulin resistance and metabolic disorders, impaired Ca^2+^ handling, mitochondrial dysfunction, oxidative stress, myocardial hypertrophy and myocardial fibrosis.

Diabetic cardiomyopathy (DCM) is a pathophysiological condition resulting from diabetes mellitus (DM) that ultimately culminates in heart failure (HF). DCM can manifest in both type 1 and type 2 diabetes, with type 2 accounting for the majority of cases (90%–95%).^4^ Among patients with DM, cardiovascular disease, especially coronary artery disease and ischemic cardiomyopathy, is the primary cause of mortality.^5^ However, DCM is defined as myocardial dysfunction in the absence of coronary artery disease, hypertension or valvular heart disease.^6^ Patients diagnosed with DCM initially present with myocardial diastolic dysfunction, which is clinically evident as HF with preserved ejection fraction.^7^, ^8^ Irrespective of the preservation or reduction in cardiac function, the most prominent features of DCM are increased extracellular matrix production and left ventricular (LV) hypertrophy.^9^, ^10^, ^11^ The pathogenesis of DCM is intricate, encompassing systemic insulin resistance and metabolic disorders, impaired Ca^2+^ handling, reduced nitric oxide bioavailability, mitochondrial dysfunction, elevated oxidative stress, myocardial fibrosis, cardiac autonomic neuropathy and microvascular dysfunction^7^ (Figure 1).

An increasing number of evidence shows a strong correlation between DCM and both genetic and environmental factors. Moreover, it has been suggested that the impaired contraction of the myocardium may be attributed, at least in part, to PTMs of specific proteins.^4^ Furthermore, the therapeutic options available for diastolic dysfunction are limited, thus emphasizing the urgency of comprehending the molecular mechanisms underlying diabetic cardiac dysfunction. This review profoundly summarizes and discusses the common PTMs implicated in the pathogenesis of DCM, including O‐GlcNAcylation, phosphorylation, methylation, acetylation and ubiquitination. The aim is to establish a novel theoretical framework and therapeutic target for the management of DCM.

## 2 PROTEIN O‐GlcNAcylation IN DIABETIC CARDIOMYOPATHY

O‐GlcNAcylation is a form of protein modification where O‐linked β‐N‐acetylglucosamine (O‐GlcNAc) is attached to serine and threonine residues.^12^ This process involves two enzymes: O‐GlcNAc transferase (OGT) and O‐GlcNAcase (OGA, also referred to as NCOAT or MGEA5).^13^, ^14^ OGT covalently attaches O‐GlcNAc to the protein from the glycosylated donor uridine diphosphate N‐acetylglucosamine (UDP‐GlcNAc, the end product of metabolic hexosamine biosynthetic pathway), which means that catalyses the transfer of O‐GlcNAc from UDP‐GlcNAc to serine and threonine, while OGA hydrolyzes O‐GlcNAc from the protein.^13^ This process is intricately linked to glucose metabolism and arises from an overload of the hexosamine biosynthesis pathway.^15^ As a result, a correlation exists between elevated O‐GlcNAcylation levels and diabetes, ultimately contributing to the pathogenesis of various cardiovascular diseases.^16^

**FIGURE 2:**
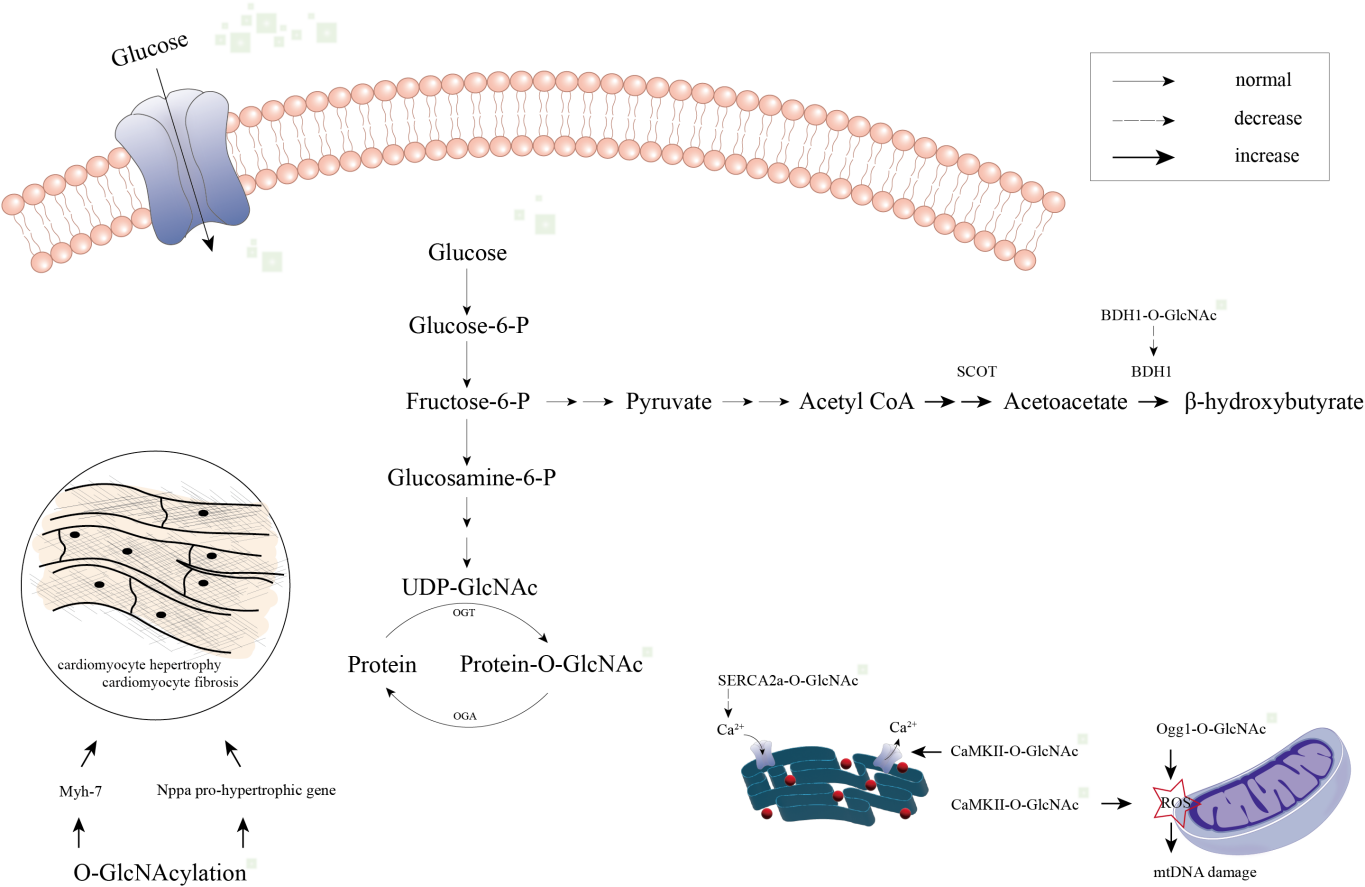
Pathogenesis associated with O‐GlcNAcylation and diabetic cardiomyopathy. The level of cardiac acetoacetate and β‐hydroxybutyrate was striking increased; BDH1 was directly modified by O‐GlcNAc and then the expression was suppressed, inhibiting ketone body utilization. In addition, SERCA2a and CaMKII can be modified by O‐GlcNAc, leading to impaired diastolic Ca^2+^ sequestering and increased sarcoplasmic reticulum Ca^2+^ release. Meanwhile, enhanced CaMKII activity by O‐GlcNAcylation induced ROS production. Elevated Ogg1 expression increased mitochondrial DNA damage. Furthermore, Myh‐7 and Nppa pro‐hypertrophic gene expression increased under O‐GlcNAcylation, presenting cardiac fibrosis.

In DCM, the primary pathophysiological manifestations are cardiac insulin resistance and subsequent metabolic disturbances. Unlike the normal adult heart, which primarily relies on fatty acid oxidation for energy production, the diabetic heart exhibits the ability to redirect acetyl coenzyme A (CoA) towards ketone body synthesis, resembling the metabolic pattern typically observed in the foetal heart.^17^, ^18^ This metabolic alteration is evidenced by the significantly elevated levels of cardiac acetoacetate and β‐hydroxybutyrate in diabetic mice^19^ (Figure 2). Acetoacetate and β‐hydroxybutyrate are the two principal ketone bodies that can be produced through the catabolism of acetyl CoA by the enzymes SCOT (succinyl‐CoA: 3‐oxoacid CoA transferase) and BDH1 (β‐hydroxybutyrate dehydrogenase).^20^ It was observed that BDH1, the enzyme responsible for β‐hydroxybutyrate dehydrogenase activity, undergoes direct modification by O‐GlcNAc, leading to suppressed expression. This finding suggests that increased O‐GlcNAcylation may hinder the utilization of ketone bodies^19^ (Figure 2). Consequently, O‐GlcNAcylation could serve as a potential mechanistic link connecting glucose and ketone body metabolism. The current consensus in academia is that acute increases in O‐GlcNAcylation have a protective effect against cardiac injury. Conversely, chronic increases in O‐GlcNAcylation have been linked to impaired calcium handling and contractile properties, mitochondrial dysfunction, myocardial hypertrophy and myocardial fibrosis, thereby exacerbating the development of DCM. The role of Ca^2+^ release and uptake by the sarcoendoplasmic reticulum in myocardial contraction and relaxation is widely recognized in academic literature. It has been established that the rate of calcium sequestration into the sarcoendoplasmic reticulum is controlled by the sarcoendoplasmic reticulum ATPase 2a (SERCA2a) gene.^21^ Notably, heightened O‐GlcNAcylation has been found to reduce the expression of the SERCA2a gene, partially through its impact on nuclear transcription factors. This process ultimately led to compromised diastolic Ca^2+^ sequestering^22^ (Figure 2). Interestingly, the presence of O‐GlcNAcylated myofilaments had a significant impact on myocardial contractility, potentially due to a decrease in cardiac myofilament Ca^2+^ sensitivity resulting from increased O‐GlcNAcylation levels.^23^ Calcium/calmodulin (Ca^2+^/CaM) dependent protein kinase II (CaMKII), a multifunctional serine/threonine kinase, is known to respond to acute β‐adrenergic activation and contribute to cardiac remodelling under pathological stress.^24^, ^25^ Numerous studies have demonstrated the involvement of CaMKII in various heart diseases, including myocardial hypertrophy,^25^ HF,^25^, ^26^ ischemia/reperfusion injury,^27^ and myocardial infarction.^28^ In recent years, research has shown that under conditions of diabetic hyperglycaemia, CaMKII can be modified by O‐GlcNAc, resulting in its activation and subsequent augmentation of sarcoplasmic reticulum Ca^2+^ release^29^ (Figure 2). Specifically, the increased CaMKII activity was consistent with O‐GlcNAcylated CaMKII S279.^29^ Meanwhile, the increased O‐GlcNAcylation, coupled with CaMKII activity, prompted the generation of reactive oxygen species (ROS) in mouse cardiomyocytes, further aggravating DCM^30^ (Figure 2). While the inhibition of CaMKII activity might restore the systolic and diastolic function of the myocardium in individuals with type 2 diabetes.^31^ In addition, O‐GlcNAcylation induced by DM may lead to mitochondrial dysfunction associated with DCM, and over 88 mitochondrial proteins can be O‐GlcNAcylated.^32^, ^33^ The main manifestation was not only a more than twofold increase in mitochondrial protein O‐GlcNAcylation in DCM with an increase in OGT and a decrease in OGA, but also mislocalized OGT in diabetic mitochondria with impaired activity of complex IV.^32^ Molecular insights implicated that elevated O‐GlcNAcylation of 8‐oxoguanine DNA glycosylase (Ogg1, the main DNA glycosylase responsible for repair of the ROS‐induced mutagenic DNA lesion) increased mitochondrial DNA damage^34^ (Figure 2). Regarding myocardial hypertrophy, previous studies have demonstrated that O‐GlcNAcylation can induce the activation of transcription factors, including NFAT (nuclear factor of activated T‐cells), GATA4 or MEF2C (myocyte enhancer factor 2C).^35^, ^36^ A recent study proved that elevated O‐GlcNAc modification is associated with cardiac fibrosis and upregulated expression of pro‐hypertrophic genes such as Myh‐7 and Nppa^37^ (Figure 2). In humans, there is a correlation between elevated O‐GlcNAcylation and LV dysfunction. Additionally, the density of LV O‐GlcNAc is positively correlated with blood glucose levels and inversely correlated with LV ejection fraction.^37^ The impaired cardiac phosphatidylinositol 3‐kinase (PI3K) (p110α)/AKT signalling is suggested as a potential mechanism behind this phenomenon. O‐GlcNAcylation often contributes to cardiac damage, but it has also been demonstrated that certain molecules modified by O‐GlcNAcylation can provide protection against HF in DCM. For instance, it was shown that the re‐expression of the N‐terminal proteolytic fragment of Histone Deacetylase 4 (HDAC4‐NT) under high O‐GlcNAc conditions can serve as a preventive measure against HDAC4‐dependent DCM, whereas mice lacking HDAC4 (HDAC4‐KO) develop HF.^38^ The effects of O‐GlcNAcylation on the heart appear to be inconsistent and depend on specific changes at certain sites.

The intricate nature of protein O‐GlcNAcylation in cardiovascular disease gives rise to highly nuanced effects that are contingent upon the specific target proteins involved. As mentioned above, modifications of different genes result in different outcomes in Ca^2+^ handling, leading to impaired myocardial contraction or relaxation function. Promising strategies for restoring physiological O‐GlcNAc homeostasis and improving cardiac function include targeted interventions at specific loci and regulatory modifications, such as adeno‐associated viral vector gene therapy.^37^

## 3 PROTEIN PHOSPHORYLATION IN DIABETIC CARDIOMYOPATHY

Phosphorylation, a well‐researched PTMs, exerts regulatory control over cell growth, differentiation, apoptosis and signal transduction. Unlike O‐GlcNAcylation, phosphorylation is governed by a multitude of specific kinases and phosphatases, responsible for the addition and removal of phosphate groups, respectively.^39^ The serine/threonine kinases comprise the largest subset of these enzymes. In contrast to O‐GlcNAcylation, phosphorylation indirectly impairs cardiac function by affecting various signalling pathways. Notably, the phosphorylation of PI3K/AKT assumes a crucial role in insulin resistance and the pathogenesis of DCM.^40^

**FIGURE 3:**
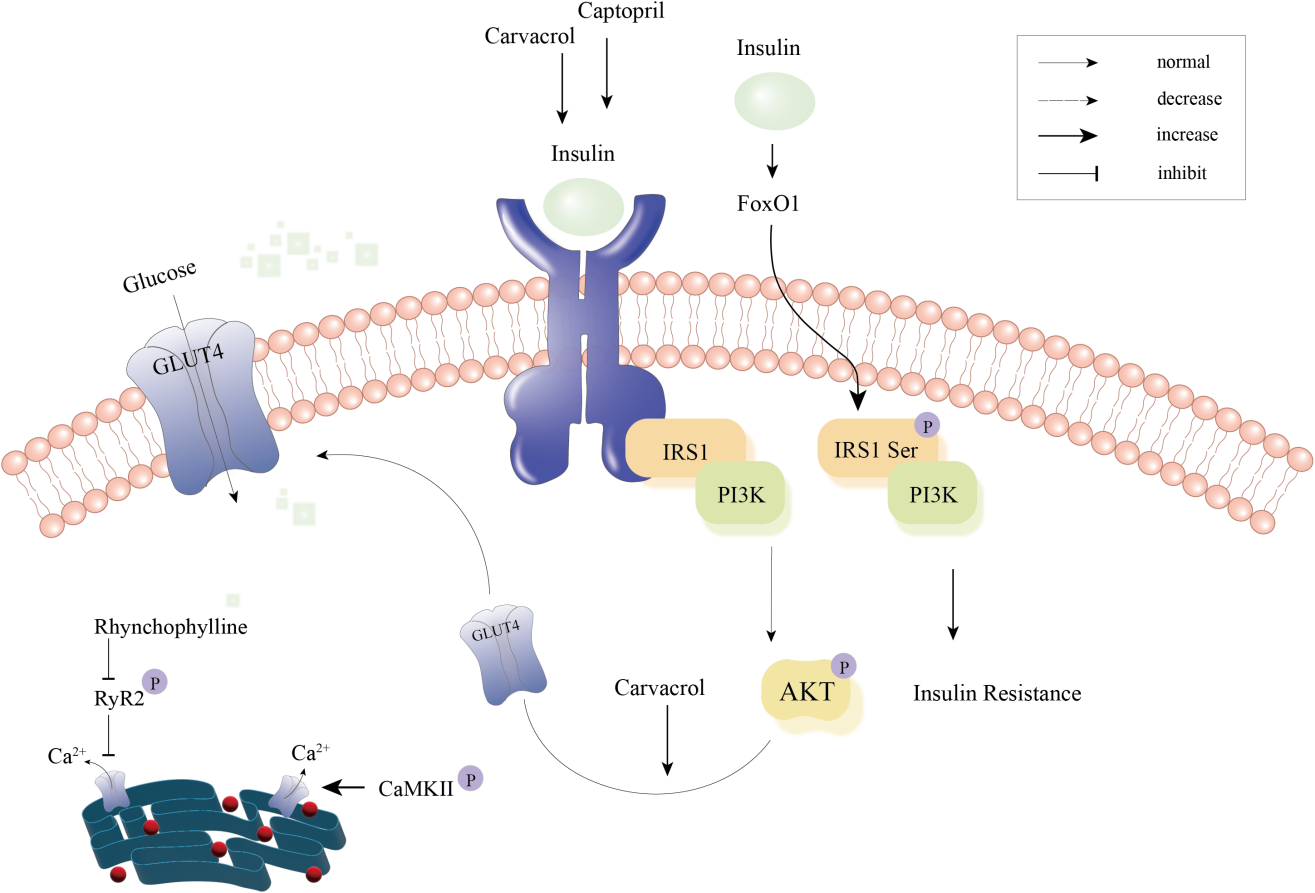
Pathogenesis associated with phosphorylation and diabetic cardiomyopathy. Under physiological conditions, upon insulin binding to the sarcolemmal insulin receptor, activation of PI3K/AKT signalling occurs upstream. This activation leads to glucose uptake by myocardial tissue through GLUT4 at the plasma membrane. Sustained activation of FoxO1 increased pIRS1 Ser levels, ultimately leading to insulin resistance. While captopril can restore insulin signalling and increase AKT phosphorylation, then normalizing substrate utilization. Carvacrol can promote the phosphorylation of PI3K and AKT, and enhance GLUT4 translocation to the cell membrane of cardiomyocytes as well. In addition, rhynchophylline can antagonize RyR2 phosphorylation to inhibit Ca^2+^ leakage.

The classical signalling cascade of the insulin receptor (IR) commences with its binding to insulin, followed by autophosphorylation of IR and subsequent activation of various kinases, including the PI3K‐AKT signalling pathway.^41^ Then, the downstream signals regulate cellular physiological processes, such as glucose transport, glycogen synthesis and protein translation by activating numerous downstream target proteins.^41^ Research demonstrated that the association between β‐adrenergic signalling and insulin resistance exhibited a biphasic effect.^42^ Short‐term stimulation increased insulin‐stimulated glucose uptake through PKA/Ca^2+^‐dependent and PI3K‐independent pathways mediated AKT phosphorylation. In contrast, long‐term stimulation resulted in the inhibition of insulin‐stimulated glucose uptake and insulin‐induced autophosphorylation of IRs in cardiac myocytes, while concurrently promoting threonine phosphorylation of IRs. Insulin resistance is additionally linked to the activation of the renin‐angiotensin system.^43^ Studies have shown that the administration of the renin‐angiotensin system inhibitor captopril could potentially rectify insulin signalling and regulate substrate utilization within the myocardium, thereby enhancing cardiac function.^44^ The underlying mechanisms involved may entail heightened phosphorylation of AKT and reduced activation of adenosine mono phosphate‐activated protein kinase (AMPK), ultimately leading to the restoration of insulin sensitivity and the improvement of myocardial energetics^44^ (Figure 3). The transcription factor forkhead box‐containing protein O (FoxO) is widely recognized as a crucial component in regulating insulin signalling, acting downstream of insulin.^45^ In the setting of systemic insulin resistance, the targeted removal of FoxO1 and FoxO3 specifically in cardiomyocytes was associated with the preservation of cardiac function.^46^ Molecular insights implicated that the sustained activation of FoxO1 contributed to the elevation of pIRS1 Ser levels, ultimately resulting in the development of insulin resistance (Figure 3). Meanwhile, FoxO1‐dependent downregulation of IRS1 led to reduced AKT signalling and insulin resistance.^46^ The insulin‐stimulated PI3K/AKT signalling pathway regulates the glucose transporter type 4 (GLUT4), which is essential for glucose uptake and metabolism.^47^ As said before, the binding of insulin to the sarcolemmal IR triggers the activation of the PI3K/AKT signalling pathways. This activation facilitated the uptake of glucose by myocardial tissue through GLUT4 located at the plasma membrane, thereby playing a critical role in sustaining cardiac energy supply^48^, ^49^ (Figure 3). In contrast, decreased GLUT4 protein expression and translocation to the cell surface in cardiomyocytes was considered to be among the pathogenic mechanisms of DCM.^49^ These observations collectively suggest the involvement of AKT in the regulation of insulin sensitivity. Carvacrol, a monoterpenic phenol isolated from various mints, exhibited notable effects on the phosphorylation of PI3K, PDK1 and AKT, while concurrently reducing PTEN phosphorylation, thereby restoring the PI3K/AKT signalling pathway^50^ (Figure 3). Of particular significance, carvacrol effectively enhanced GLUT4 translocation to the cell membrane of cardiomyocytes, exerting an anti‐DCM effect^50^ (Figure 3). Additionally, Chinese herbal Rhynchophylline (Rhy) has the anti‐DCM effect as well. Molecular insights implicated that Rhy can inhibit the overactivation of the sarcoplasmic reticulum Ryanodine receptor 2 (RyR2), which was known to cause increased Ca^2+^ leakage. This inhibition was primarily achieved by antagonizing RyR2 phosphorylation thereby regulating calcium homeostasis^51^ (Figure 3). As mentioned previously, CaMKII Ser280 can be activated by O‐GlcNAcylation to release Ca^2+^ and, in addition, CaMKII can be activated by phosphorylation^52^ (Figure 3). There is also cross‐talking between phosphorylation and ubiquitination. For instance, Y‐box binding protein‐1 (YB‐1), a member of the highly conserved cold shock domain protein family, exacerbated DCM when its total protein levels were significantly reduced, accompanied by elevated protein phosphorylation levels.^53^ The stability of YB‐1 was regulated by phosphorylation, which facilitated its degradation via otubain‐1 (OTUB1)‐dependent ubiquitination. This mechanism was mediated by the upstream ERK/RSK signalling pathway, and inhibition of the ERK pathway can ameliorate DCM.^53^ In relation to myocardial fibrosis, it was hypothesized that Sal B, the active components of *Salvia miltiorrhiza*, could enhance cardiac function in mice with DCM by attenuating hyperglycemia‐induced myocardial remodelling and myocardial fibrosis.^54^, ^55^ Mechanistically, Sal B was found to augment the phosphorylation of AKT and ERK by inhibiting the expression of insulin‐like growth factor‐binding protein 3 (IGFBP3), activating these signalling pathway and thus promoting angiogenesis.^55^

In brief, the regulation of glucose metabolism is significantly influenced by the involvement of PI3K/AKT. The diminished phosphorylation and activation of PI3K and AKT hinder the insulin‐mediated glucose uptake, exerting a substantial effect on insulin actions within the cardiac system. Site‐specific targeted activation of AKT phosphorylation may play a role in alleviating insulin resistance and potentially reversing DCM. Given that phosphorylation is regulated by the competing activities of protein kinases and phosphatases,^56^ a considerable array of pharmaceutical agents drugs targeting protein kinases/phosphatases has been devised. The ROCK kinases (Rho‐associated coiled‐coil containing kinases), which belong to the serine/threonine protein kinase family, exert a crucial influence on regulating the actin cytoskeleton to influence cell motility and regulate vascular tone.^57^ Fasudil, a ROCK inhibitor, has demonstrated efficacy in ameliorating symptoms associated with cardiovascular conditions, including hypertension, angina pectoris and ischemic stroke, among others.^58^ In addition, CP‐91149, an inhibitor of glycogen phosphorylase, has been studied in type 2 diabetes to increase glucose availability and meet high energy demands.^59^

## 4 PROTEIN METHYLATION IN DIABETIC CARDIOMYOPATHY

As an important epigenetic regulation, methylation refers to the transfer of active methyl groups into target chemicals, catalysed by methyltransferases, to form methylation products. The process is reversible and involves two enzymes, methyltransferase and demethylase. Methylation includes DNA methylation, RNA methylation and protein methylation (subdivided into histone and non‐histone methylation).^60^, ^61^, ^62^, ^63^ In cardiovascular disease, the role of methylation has been recognized and relevant preclinical studies and drugs targeting DNA methyltransferases and histone methyltransferases have emerged.^64^ In this review, we mainly discuss histone methylation, which usually occurs at the arginine and lysine residues of histone 3 (H3) and histone 4 (H4) and affects the transcriptional activity of related genes.

**FIGURE 4:**
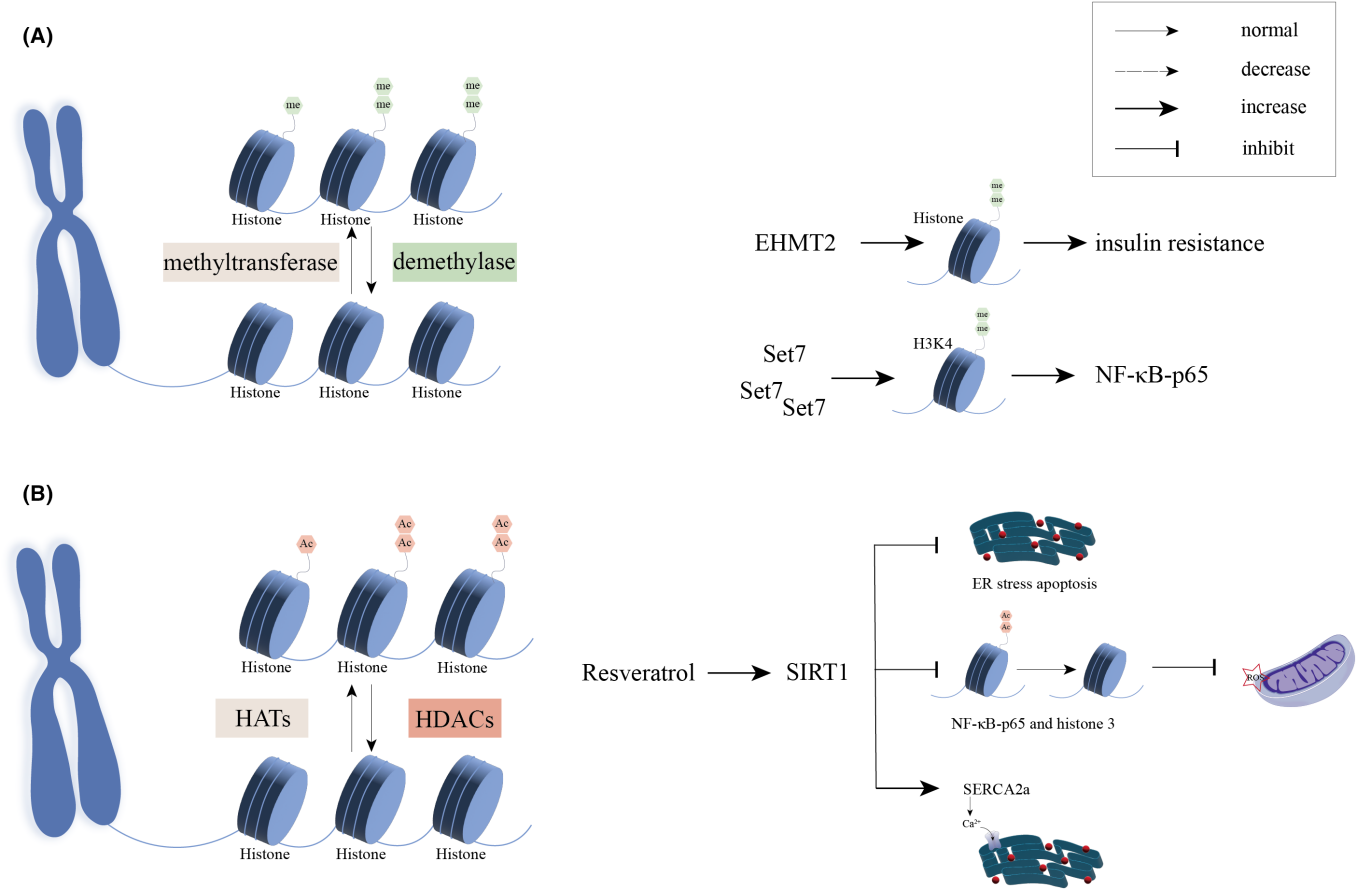
Pathogenesis associated with methylation and acetylation and diabetic cardiomyopathy. (A) EHMT2 mediated histone methylation can cause insulin resistance. The recruitment of the methyltransferase Set7 led to an increase in H3K4 methylation, which resulted in a sustained increase in NF‐κB‐p65 gene expression. (B) Resveratrol could activate SIRT1 and alleviate endoplasmic reticulum stress‐mediated apoptosis; resveratrol could ameliorate oxidative stress through deacetylation of NF‐κB‐p65 and histone 3; resveratrol could upregulate SERCA2a expression.

Insulin resistance and metabolic disorders are fundamental parts in the development of DCM. Decreased EHMT2 (euchromatic histone lysine methyltransferase 2) in the liver of leptin receptor gene‐deficient mice can downregulate HMGA1 gene expression, impairing IR transcription.^65^ It has been reported to cause insulin resistance in the liver, which in turn led to systemic insulin resistance (Figure 4A). Classical nuclear factor kappa‐B (NF‐κB) plays a crucial role in the immune response, and activation of NF‐κB‐p65 is also a feature of DCM. Under high glucose induction, the recruitment of the methyltransferase Set7 led to an increase in H3K4 methylation, which resulted in a sustained increase in NF‐κB‐p65 gene expression (Figure 4A). And this process may be caused by the release of ROS from exposure to high glucose.^66^, ^67^ Moreover, in a diabetic mouse model, renal failure increased dimethylation of cardiac histone H3K4, which promoted cardiomyocyte hypertrophy and exacerbated DCM.^68^ In DCM, the mechanisms underlying histone methylation lack specificity, and targeted intervention studies are currently lacking. However, several studies have demonstrated that modulating histone methylation modifications in adipocytes can effectively inhibit lipid storage capacity.^69^, ^70^, ^71^ Lipid deposition can damage myocardial calmodulin, which in turn affects the diastolic and systolic functions of cardiomyocytes.^72^ Exploring the potential impact of such interventions in cardiomyocytes may offer a therapeutic approach to ameliorate insulin resistance and metabolic disorders. Intervening in the response to histone methylation‐induced inflammation is also one of the strategies.^73^ Galangin can ameliorate DCM by reducing oxidative stress and inflammation.^74^, ^75^ Hao et al. proposed that DNA methylation and histone modifications may play an important role in microvascular complications of DCM and thereby represent a potential therapeutic target.^76^

## 5 PROTEIN ACETYLATION IN DIABETIC CARDIOMYOPATHY

Acetylation is the process of transferring an acetyl group to an amino acid side‐chain group, with histone acetylation being the most prevalent form.^77^ Histone acetylation is mediated by coactivator complexes containing histone acetyltransferases (HATs), while deacetylation is mediated by co‐repressor complexes containing HDACs.^77^ In addition to this, acetylation can also occur at the non‐histone or N‐terminal regions of proteins. Histone acetyltransferases can be classified into four major groups based on their structure and properties, namely CBP/p300, GCN5, MYST and SRC/p160, while the NAD^+^‐dependent sirtuin (SIRT) family represents the most prevalent deacetylase.^78^ Extensive research has demonstrated the crucial role of acetylation and deacetylation of functional proteins in cardiomyocyte differentiation, cardiac remodelling and various cardiovascular disorders, such as DCM.

The reduced NAD+/NADH ratio and consequent inactivation of sirtuins have been identified as contributing factor to the decreased cardiac metabolic rate observed in individuals with diabetes.^79^ Numerous studies have been conducted on HDACs, revealing the regulatory roles of SIRT1 and SIRT3 in DCM through their influence on cardiomyocyte metabolism. Notably, SIRT3 overexpression attenuated hyperglycemia‐induced glycolysis and oxidative stress damage by inhibiting p53 acetylation and TP53‐induced expression of glycolytic and apoptotic regulators.^80^ Conversely, the impact of SIRT1 on cardiac function appeared to be contingent upon the dosage administered. Specifically, high doses of SIRT1 induced cardiomyopathy, whereas mild to moderate levels of SIRT1 expression did not exhibit this adverse effect.^81^ Furthermore, even though high expression of SIRT1 increased oxidative stress, moderate SIRT1 induced resistance to oxidative stress and apoptosis. Resveratrol could activate SIRT1 and alleviate endoplasmic reticulum stress‐mediated apoptosis in DCM (Figure 4B), and PERK/eIF2α, ATF6/CHOP and IRE1α/JNK signalling pathways may be involved in this process.^82^, ^83^ According to Bagul et al., in the presence of resveratrol, SIRT1 ameliorated oxidative stress in DCM through deacetylation of NF‐κB‐p65 and histone 3^84^ (Figure 4B). Sulaiman et al. suggested that resveratrol adjusted calcium homeostasis to improve cardiac function by activating SIRT1 and upregulating SERCA2a expression^85^ (Figure 4B). Therefore, SIRT1 is considered as a potential target for the treatment of cardiovascular diseases, especially DCM.^86^ Resveratrol can activate SIRT2, SIRT3 and SIRT5 as well, which played an indispensable role in diabetic cardiomyopathy.^87^ Transforming growth factor beta (TGF‐β) is a pro‐sclerotic cytokine that is associated with cardiac fibrosis and hypertrophy.^88^ Under high glucose conditions, p300 activity was increased, while p300 enhanced TGF‐β activity via Smad2 acetylation, which was involved in fibrotic interstitium.^89^ Hyperglycemia‐induced advanced glycation end products (AGEs) impaired Na^+^‐K^+^‐ATPase activity by decreasing AMPK and SIRT1, leading to contractile dysfunction of the heart.^90^, ^91^ Meanwhile, further SIRT1‐downregulation enhanced endothelial cell death, one of the main factors of the increased endothelial permeability in DM.^92^ It was mentioned in the section on phosphorylation modifications that reduced glucose uptake in the hearts of mice with DCM was associated with decreased expression of GLUT1 and GLUT4, as well as a decrease in insulin‐stimulated GLUT4 translocation to the sarcolemma.^49^ HDAC inhibition can upregulate the GLUT1 and GLUT4 expression, accompanied by increased GLUT1 acetylation and p38 phosphorylation.^93^ It can not only reduce cardiac hypertrophy but also increase angiogenesis, ultimately improving cardiac function. HDAC knockout diabetic mice developed HF. However, the re‐expression of the HDAC4 N‐terminal fragment prevented HDAC4‐dependent DCM. The production of the HDAC4 N‐terminal fragment relied primarily on dependent on PTMs of HDAC4 by O‐GlcNAcylization at serine (Ser)‐642 to counteract pathological CaMKII signalling.^38^

Currently, there are many studies on acetylation affecting the mitochondrial NAD^+^/NADH redox state. Berthiaume et al. proposed a method based on alternate mitochondrial electron transport that normalizes the mitochondrial redox state and improves DCM.^94^ Dietary NAD+ supplementation or pharmacological activation of sirtuins may restore the NAD+/NADH ratio, improving mitochondrial function and cardiac performance.^95^, ^96^ In addition, exercise has been shown to have pleiotropic benefits on cardiometabolic homeostasis as a strategy for non‐pharmacological treatment. Treadmill exercise significantly attenuated diabetes‐induced cardiac insufficiency, accompanied by reduced mitochondrial damage and increased cardiac mitochondrial enzyme activity. Molecular insights implicated that the exercise response factor, fibroblast growth factor 21 (FGF21), maintained normal mitochondrial function by inducing the AMPK/FoxO3/SIRT3 signalling axis, thereby reversing diabetes‐induced hyperacetylation and dysfunction of the mitochondrial enzyme cluster.^97^

## 6 PROTEIN UBIQUITINATION IN DIABETIC CARDIOMYOPATHY

Ubiquitin is a low molecular weight protein with 76 amino acid residues that can be tagged on the surface of a specific protein substrate by the action of enzymes and then recognized and degraded by organelles or multi‐enzyme complexes.^98^ The ubiquitin proteasome pathway refers to the degradation of ubiquitinated target proteins by the 26S proteasome in an ATP‐dependent manner.^99^ The process is specifically modified by a cascade reaction of three enzymes, including E1 ubiquitin activating enzyme, E2 ubiquitin binding enzyme and E3 ubiquitin ligase.^100^ There are more than 600 types of E3 ubiquitin ligases that have been identified. The classical E3 ubiquitin ligases are divided into three main types, RING (Really Interesting New Gene) type, HECT (Homologous to E6AP C‐terminus) type and RBR (RING‐between‐RING) type.^101^ The specific recognition of substrates by E3 ubiquitin ligases makes their role in the ubiquitin pathway particularly important and much more studied.

**FIGURE 5:**
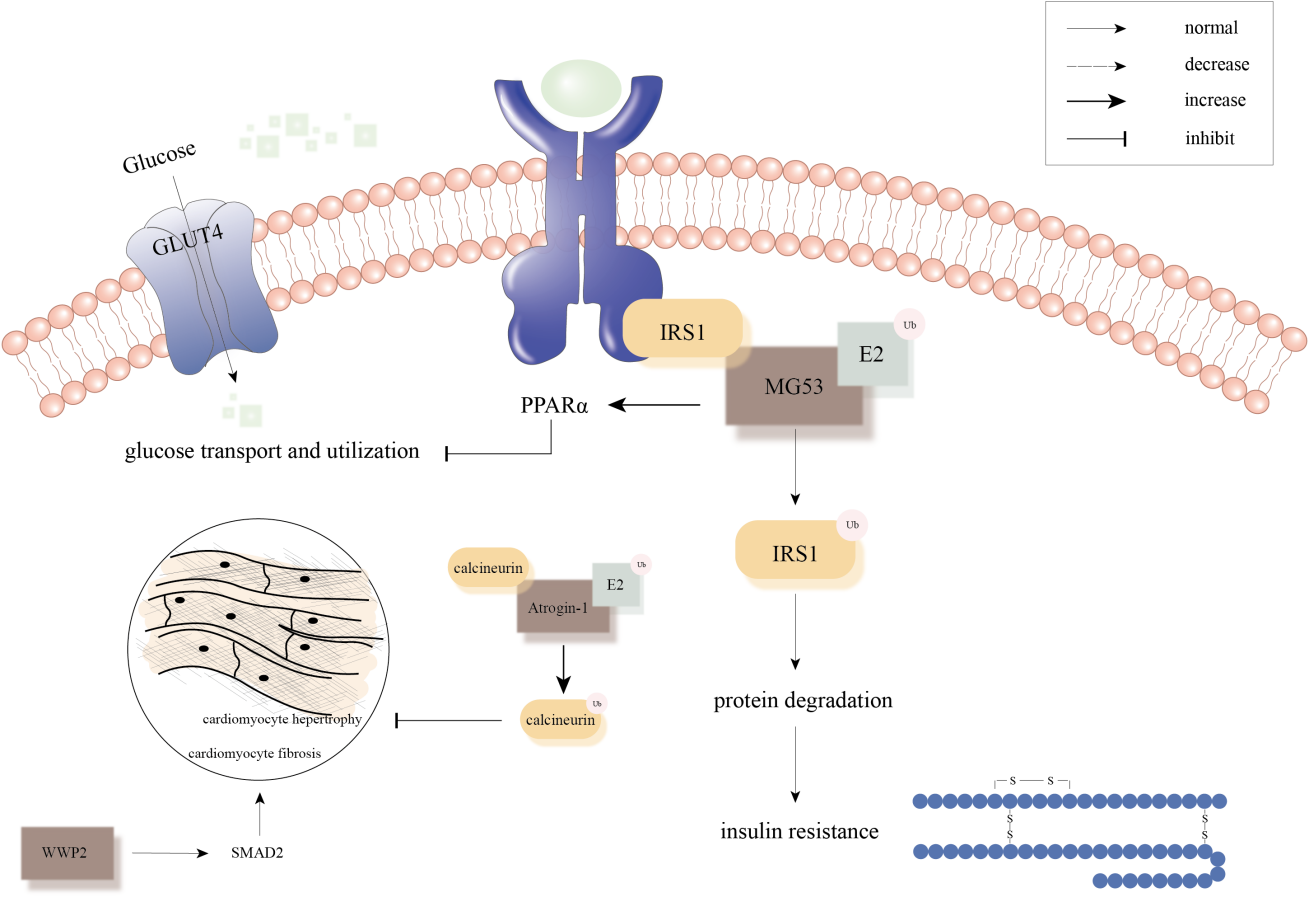
Pathogenesis associated with ubiquitination and diabetic cardiomyopathy. MG53 can upregulate PPARα expression, decreasing genes involved in glucose transport and utilization. While inhibition of MG53 mediated ubiquitination degradation of IRS‐1 contributed to amelioration of insulin resistance. Atrogin‐1 promoted ubiquitination of calcineurin, thereby inhibiting cardiomyocyte hypertrophy. WWP2 can regulate cardiac fibrosis by modulating SMAD2 signalling.

Several E3 ligases are involved in the development of insulin resistance through different pathways. One mechanism is the direct degradation of IRs (especially hyperglycemia‐induced) and related substrates via the ubiquitin proteasome pathway.^102^ For example, IR substrate (IRS) can exert biological effects by tyrosine phosphorylation in response to insulin and insulin‐like growth factor (Figure 5). Mice lacking IRS1 or IRS2 exhibited peripheral insulin resistance.^103^ Inhibition of E3 ubiquitin ligase MG53 (also known as tripartite motif‐containing 72, TRIM72) mediated ubiquitination degradation of IRS‐1 contributed to amelioration of insulin resistance^104^ (Figure 5). E3 ubiquitin ligase, Cullin7, regulated insulin signalling through the mTOR‐S6K‐IRS1 signalling axis, as evidenced by enhanced insulin sensitivity in Cullin7^+/−^ or Fbxw8^+/−^ mice.^105^ Fbxw8 is an essential component of Cullin7 and is responsible for substrate specific recognition.^106^ Insulin resistance is always presented with lipid overload, causing mitochondrial dysfunction and myocardial toxicity.^107^ Lipid overload increased AKAP121 (A kinase anchoring protein 121, a mitochondrial outer membrane scaffold protein) ubiquitination, modulated DRP1 (dynamin related protein 1, mitochondrial dynamic regulation protein) phosphorylation and altered OPA1 (optic atrophy 1, mitochondrial dynamic regulation protein) processing, eventually resulting in impaired mitochondrial energetics.^107^, ^108^, ^109^, ^110^ This process may be mediated by ROS production. In addition to regulating metabolism through target protein degradation via the ubiquitin proteasome pathway, E3 ubiquitin ligases are involved in the regulation of gene transcription.^111^ These transcription factors include PPARα (peroxisome proliferators‐activated receptor α), FOX‐O, Nrf2 (nuclear factor derived‐2) and NF‐κB.^112^, ^113^ PPARα was the first transcription factor identified to be associated with DCM.^114^ In mice with cardiac specific PPARα overexpression, expression of target genes involved in cardiac fatty acid uptake and oxidation pathways was increased, while expression of genes involved in glucose transport and utilization was decreased.^114^ Consistently, these mice had increased rates of myocardial fatty acid oxidation and decreased glucose uptake and oxidation, similar to the metabolic phenotype of the DCM.^114^ MG53 can positively upregulate the expression level of PPARα, which triggers a cascade of events leading to DCM (Figure 5). E3 ubiquitin ligases are also involved in the pathogenesis of cardiac hypertrophy and myocardial fibrosis. Atrogin‐1 (also named Muscle‐atrophy F‐box), an F‐box protein with skeletal and cardiac muscle specificity, is a component of the SCF family of E3 ubiquitin ligases.^115^ Atrogin‐1 promoted ubiquitination of calcineurin, thereby inhibiting cardiomyocyte hypertrophy^116^ (Figure 5). According to Ye et al., the deubiquitinating enzyme ubiquitin‐specific peptidase 25 (USP25) can combined with SERCA2a and prevent proteasomal pathway from degrading it.^117^ This process not only maintained calcium homeostasis in cardiomyocytes but also contributed to the inhibition of cardiac hypertrophy. Moreover, increased E3 ubiquitin ligase WWP2 expression was related with an elevated pro‐fibrotic gene expression program in diseased heart. WWP2, particularly the WWP2‐N isoform containing the N‐terminal C2 and WW1 domains of WWP2, regulated cardiac fibrosis by modulating SMAD2 signalling^118^ (Figure 5).

Currently, therapeutic approaches targeting the ubiquitin proteasome or E3 ubiquitin ligase are advancing, and targeting E3 ubiquitin ligase is considered to offer higher specificity and less toxicity compared to E1 and E2. As a proteasome inhibitor, MG132 can reverse pathogenic cardiac manifestations, including myocardial hypertrophy, fibrosis and reduced LV ejection fraction.^113^ Molecular insights suggest that MG132 increased expression of Nurf2 and downstream antioxidant gene, while decreased nuclear accumulation of cardiac NF‐κB and its DNA binding activity.^112^ The small molecule MyoMed‐205 can target MuRF1 and improve the diastolic dysfunction of the myocardium.^119^ Alongside agonists and inhibitors, PROTAC (proteolysis targeting chimaera) technology has garnered considerable attention and hold promise for the development of more specific therapeutic regimens.

## 7 DISCUSSION

In summary, the widespread and dynamic nature of PTMs implicates their involvement in various aspects of the pathogenesis of DCM, with different types of PTMs interacting to coordinate the intricate pathophysiological functions. PTMs at different sites of the same protein may have different effects on the disease. Meanwhile, because of the cross‐talk between post‐translational modifications, it is difficult to target a single specific modification to achieve a better therapeutic effect. This highlights the importance of clear mechanisms. We found that in DCM, insulin resistance and Ca^2+^ handling disorders seem to be more involved or more studied (Table 1). For one thing, insulin resistance and metabolic disorders play a central role in DCM development. Insulin resistance in the heart disrupts insulin signalling‐mediated substrate utilization, increasing the likelihood of cardiac insufficiency.^120^ Diabetic patients exhibit increased cardiac fatty acid utilization, changed glucose uptake and oxidation and increased fructose content, which may lead to mitochondrial structural remodelling and accelerate O‐GlcNAylation and AGEs formation.^107^, ^121^, ^122^, ^123^ Besides, a number of PTMs can alter the function of the mitochondrial permeability transition pore (mPTP) opening, affect energy metabolism and indirectly participate in the pathophysiology process of DCM.^124^ Among the various PTMs, phosphorylation and acetylation are overwhelmingly dominant.^124^ Dichloroacetate has shown promise in restoring myocardial substrate selection balance by enhancing pyruvate dehydrogenase flux, thereby reversing diastolic dysfunction in DCM.^125^ HDAC inhibitors, such as chidamide, have received a lot of attention in the treatment of haematological diseases, especially peripheral T‐cell lymphoma.^126^ HDAC inhibitors are also currently thought to relieve systemic insulin resistance and glucose handling,^93^, ^127^ while further studies are still needed regarding the specific regulation of HDAC on the heart. Therefore, normalizing the metabolic profile in DCM by modulating PTMs may be an important target to improve diabetic cardiac function.

**TABLE 1:** Involvement of post‐translational modifications in insulin resistance and impaired Ca^2+^ handling impacts diabetic cardiomyopathy.

PTMs	Related mechanism	
	Insulin resistance & metabolic disorders	Impaired Ca^2+^ handling
O‐GlcNAcylation	BDH1 can be modified by O‐GlcNAc and then the expression was suppressed, then inhibiting ketone body utilization^19^	O‐GlcNAcylation decreased SERCA2a gene expression, leading to impaired diastolic Ca^2+^ sequestering^22^ CaMKII can be modified by O‐GlcNAc, leading to CaMKII activation and increased sarcoplasmic reticulum Ca^2+^ release^29^
Phosphorylation	Long‐term β‐adrenergic signalling resulted in the inhibition of insulin‐stimulated glucose uptake and insulin‐induced autophosphorylation of insulin receptors^43^ Captopril can increase AKT phosphorylation and decrease AMPK activation, restoring insulin sensitivity and normalizing substrate utilization^44^ Sustained activation of FoxO1 increased pIRS1 Ser levels, leading to insulin resistance^46^	Rhynchophylline can antagonize RyR2 phosphorylation to regulate Ca^2+^ homeostasis^51^ CaMKII can be activated by phosphorylation, leading to increased sarcoplasmic reticulum Ca^2+^ release^29^
Methylation	EHMT2 can cause insulin resistance in the liver, then leads to systemic insulin resistance^65^	–
Acetylation	SIRT3 overexpression attenuated hyperglycemia‐induced glycolysis by inhibiting p53 acetylation^81^	Re‐expression of the HDAC4 N‐terminal fragment counteract pathological CaMKII signaling^38^ Resveratrol adjusted Ca^2+^ homeostasis by activating SIRT1 and upregulating SERCA2a expression^86^ β‐lap exerts cardio‐protective effects at least partially through SIRT1‐mediated SERCA2a deacetylation^125^
Ubiquitination	Inhibition of MG53 mediated ubiquitination degradation of IRS‐1 contributed to amelioration of insulin resistance^102^ Cullin7 regulated insulin signal through the mTOR‐S6K‐IRS1 signalling axis, as evidenced by enhanced insulin sensitivity in Cullin7^+/−^ or Fbxw8^+/−^ mice^103^ MG53 can upregulate the PPARα expression, with increased rates of myocardial fatty acid oxidation and decreased glucose uptake and oxidation^108^	USP25 can prevent SERCA2a ubiquitination degradation, maintaining Ca^2+^ homeostasis^111^

For another, impaired calcium handling emerges as a critical feature of DCM. In normal conditions, 80%–90% of intracellular calcium is released from the sarcoplasmic reticulum, while 10%–20% enters through L‐shaped calcium channels.^128^ The dynamic balance of sarcoplasmic reticulum calcium involves release mediated by RyR2 receptors and return mediated by SERCA ATP consumption, respectively.^129^, ^130^ Various PTMs related to calcium handling disturbance, especially O‐GlcNAcylation, contributing to prolonged diastolic SERCA2‐mediated calcium removal from the cytoplasm, increased RyR2‐mediated calcium leakage from the sarcoplasmic reticulum and alterations in myofilament calcium sensitivity. In DCM, the SERCA2a expression and activity are reduced, whereas the acetylation level is significantly increased, which is also a feature of heart failure. This alteration may be associated with increased levels of O‐GlcNAcylation and decreased levels of SIRT1. With the exception of the SIRT1 activator resveratrol, studies have shown that β‐lap (a quinone‐containing natural compound) exerts cardio‐protective effects at least partially through SIRT1‐mediated SERCA2a deacetylation.^131^, ^132^ Regarding RyR2, phosphorylation of it may contribute to DCM while causing inflammatory responses.^133^ Tian et al. proposed that interactions among RyR1, RyR2, Ca^2+^ signalling and immune‐related molecules may be relevant to DCM.^133^ Although RyR1 is expressed at relatively low levels in the heart, it appears to be a starting point in this interaction. Recent clinical studies have reported that cardiac resynchronization therapy responders exhibit a significantly reduced RyR1 glycation in peripheral lymphocytes.^134^ Thus, modulating PTMs to restore calcium homeostasis holds significant therapeutic potential for DCM.

## AUTHOR CONTRIBUTIONS

**Zhi Li:** Writing – original draft (lead). **Jie Chen:** Writing – original draft (lead). **Hailong Huang:** Writing – original draft (supporting). **Qianru Zhan:** Writing – original draft (supporting). **Fengzhi Wang:** Writing – original draft (supporting). **Zihan Chen:** Writing – review and editing (equal). **Xinwei Lu:** Writing – review and editing (equal). **Guozhe Sun:** Project administration (lead).

## FUNDING INFORMATION

The study is supported by the Natural Science Foundation of Liaoning Province of China (2022‐MS‐213).

## CONFLICT OF INTEREST STATEMENT

The authors declare no competing interests.

## References

[1] Lee JM , Hammarén HM , Savitski MM , Baek SH . Control of protein stability by post‐translational modifications. Nat Commun. 2023;14(1):201. 36639369 10.1038/s41467-023-35795-8 PMC9839724

[2] Keenan EK , Zachman DK , Hirschey MD . Discovering the landscape of protein modifications. Mol Cell. 2021;81(9):1868‐1878. 33798408 10.1016/j.molcel.2021.03.015 PMC8106652

[3] Chen L , Liu S , Tao Y . Regulating tumor suppressor genes: post‐translational modifications. Signal Transduct Target Ther. 2020;5(1):90. 32532965 10.1038/s41392-020-0196-9 PMC7293209

[4] Dillmann WH . Diabetic cardiomyopathy. Circ Res. 2019;124(8):1160‐1162. 30973809 10.1161/CIRCRESAHA.118.314665 PMC6578576

[5] Schmidt AM . Diabetes mellitus and cardiovascular disease. Arterioscler Thromb Vasc Biol. 2019;39(4):558‐568. 30786741 10.1161/ATVBAHA.119.310961 PMC6532416

[6] Maack C , Lehrke M , Backs J , et al. Heart failure and diabetes: metabolic alterations and therapeutic interventions: a state‐of‐the‐art review from the Translational Research Committee of the Heart Failure Association‐European Society of Cardiology. Eur Heart J. 2018;39(48):4243‐4254. 30295797 10.1093/eurheartj/ehy596 PMC6302261

[7] Jia G , Hill MA , Sowers JR . Diabetic cardiomyopathy: an update of mechanisms contributing to this clinical entity. Circ Res. 2018;122(4):624‐638. 29449364 10.1161/CIRCRESAHA.117.311586 PMC5819359

[8] Seferović PM , Paulus WJ . Clinical diabetic cardiomyopathy: a two‐faced disease with restrictive and dilated phenotypes. Eur Heart J. 2015;36(27):1718‐1727. 25888006 10.1093/eurheartj/ehv134

[9] Heerebeek L , Borbély A , Niessen HWM , et al. Myocardial structure and function differ in systolic and diastolic heart failure. Circulation. 2006;113(16):1966‐1973. 16618817 10.1161/CIRCULATIONAHA.105.587519

[10] Heerebeek L , Hamdani N , Handoko ML , et al. Diastolic stiffness of the failing diabetic heart: importance of fibrosis, advanced glycation end products, and myocyte resting tension. Circulation. 2008;117(1):43‐51. 18071071 10.1161/CIRCULATIONAHA.107.728550

[11] Gonzalez‐Quesada C , Cavalera M , Biernacka A , et al. Thrombospondin‐1 induction in the diabetic myocardium stabilizes the cardiac matrix in addition to promoting vascular rarefaction through angiopoietin‐2 upregulation. Circ Res. 2013;113(12):1331‐1344. 24081879 10.1161/CIRCRESAHA.113.302593 PMC4408537

[12] Holt GD , Snow CM , Senior A , et al. Nuclear pore complex glycoproteins contain cytoplasmically disposed O‐linked N‐acetylglucosamine. J Cell Biol. 1987;104(5):1157‐1164. 3571327 10.1083/jcb.104.5.1157 PMC2114481

[13] Haltiwanger RS , Holt GD , Hart GW . Enzymatic addition of O‐GlcNAc to nuclear and cytoplasmic proteins. Identification of a uridine diphospho‐N‐acetylglucosamine: peptide beta‐N‐acetylglucosaminyltransferase. J Biol Chem. 1990;265(5):2563‐2568. 2137449

[14] Dong DL , Hart GW . Purification and characterization of an O‐GlcNAc selective N‐acetyl‐beta‐D‐glucosaminidase from rat spleen cytosol. J Biol Chem. 1994;269(30):19321‐19330. 8034696

[15] Gonzalez‐Rellan MJ , Fondevila MF , Dieguez C , Nogueiras R . O‐GlcNAcylation: a sweet hub in the regulation of glucose metabolism in health and disease. Front Endocrinol (Lausanne). 2022;13:873513. 35527999 10.3389/fendo.2022.873513 PMC9072661

[16] Bolanle IO , Riches‐Suman K , Williamson R , Palmer TM . Emerging roles of protein O‐GlcNAcylation in cardiovascular diseases: insights and novel therapeutic targets. Pharmacol Res. 2021;165:105467. 33515704 10.1016/j.phrs.2021.105467

[17] Jong KAD , Lopaschuk GD . Complex energy metabolic changes in heart failure with preserved ejection fraction and heart failure with reduced ejection fraction. Can J Cardiol. 2017;33(7):860‐871. 28579160 10.1016/j.cjca.2017.03.009

[18] Cook GA , Lavrentyev EN , Pham K , Park EA . Streptozotocin diabetes increases mRNA expression of ketogenic enzymes in the rat heart. Biochim Biophys Acta Gen Subj. 2017;1861(2):307‐312. 27845231 10.1016/j.bbagen.2016.11.012 PMC5362824

[19] Brahma MK , Ha C , Pepin ME , et al. Increased glucose availability attenuates myocardial ketone body utilization. J Am Heart Assoc. 2020;9(15):e013039. 32750298 10.1161/JAHA.119.013039 PMC7792234

[20] Puchalska P , Crawford PA . Multi‐dimensional roles of ketone bodies in fuel metabolism, signaling, and therapeutics. Cell Metab. 2017;25(2):262‐284. 28178565 10.1016/j.cmet.2016.12.022 PMC5313038

[21] Periasamy M , Huke S . SERCA pump level is a critical determinant of Ca(2+)homeostasis and cardiac contractility. J Mol Cell Cardiol. 2001;33(6):1053‐1063. 11444913 10.1006/jmcc.2001.1366

[22] Clark RJ , McDonough PM , Swanson E , et al. Diabetes and the accompanying hyperglycemia impairs cardiomyocyte calcium cycling through increased nuclear O‐GlcNAcylation. J Biol Chem. 2003;278(45):44230‐44237. 12941958 10.1074/jbc.M303810200

[23] Ramirez‐Correa GA , Jin W , Wang Z , et al. O‐linked GlcNAc modification of cardiac myofilament proteins: a novel regulator of myocardial contractile function. Circ Res. 2008;103(12):1354‐1358. 18988896 10.1161/CIRCRESAHA.108.184978 PMC2615199

[24] Grimm M , Brown JH . Beta‐adrenergic receptor signaling in the heart: role of CaMKII. J Mol Cell Cardiol. 2010;48(2):322‐330. 19883653 10.1016/j.yjmcc.2009.10.016 PMC2896283

[25] Anderson ME , Brown JH , Bers DM . CaMKII in myocardial hypertrophy and heart failure. J Mol Cell Cardiol. 2011;51(4):468‐473. 21276796 10.1016/j.yjmcc.2011.01.012 PMC3158288

[26] Bossuyt J , Helmstadter K , Wu X , et al. Ca2+/calmodulin‐dependent protein kinase IIdelta and protein kinase D overexpression reinforce the histone deacetylase 5 redistribution in heart failure. Circ Res. 2008;102(6):695‐702. 18218981 10.1161/CIRCRESAHA.107.169755

[27] Ling H , Gray CBB , Zambon AC , et al. Ca2+/Calmodulin‐dependent protein kinase II δ mediates myocardial ischemia/reperfusion injury through nuclear factor‐κB. Circ Res. 2013;112(6):935‐944. 23388157 10.1161/CIRCRESAHA.112.276915 PMC3673710

[28] Weinreuter M , Kreusser MM , Beckendorf J , et al. CaM Kinase II mediates maladaptive post‐infarct remodeling and pro‐inflammatory chemoattractant signaling but not acute myocardial ischemia/reperfusion injury. EMBO Mol Med. 2014;6(10):1231‐1245. 25193973 10.15252/emmm.201403848 PMC4287929

[29] Erickson JR , Pereira L , Wang L , et al. Diabetic hyperglycaemia activates CaMKII and arrhythmias by O‐linked glycosylation. Nature. 2013;502(7471):372‐376. 24077098 10.1038/nature12537 PMC3801227

[30] Lu S , Liao Z , Lu X , et al. Hyperglycemia acutely increases cytosolic reactive oxygen species via O‐linked GlcNAcylation and CaMKII activation in mouse ventricular myocytes. Circ Res. 2020;126(10):e80‐e96. 32134364 10.1161/CIRCRESAHA.119.316288 PMC7210078

[31] Daniels LJ , Wallace RS , Nicholson OM , et al. Inhibition of calcium/calmodulin‐dependent kinase II restores contraction and relaxation in isolated cardiac muscle from type 2 diabetic rats. Cardiovasc Diabetol. 2018;17(1):89. 29903013 10.1186/s12933-018-0732-x PMC6001139

[32] Banerjee PS , Ma J , Hart GW . Diabetes‐associated dysregulation of O‐GlcNAcylation in rat cardiac mitochondria. Proc Natl Acad Sci U S A. 2015;112(19):6050‐6055. 25918408 10.1073/pnas.1424017112 PMC4434690

[33] Ma J , Liu T , Wei A , et al. O‐GlcNAcomic profiling identifies widespread O‐linked β‐N‐Acetylglucosamine modification (O‐GlcNAcylation) in oxidative phosphorylation system regulating cardiac mitochondrial function. J Biol Chem. 2015;290(49):29141‐29153. 26446791 10.1074/jbc.M115.691741 PMC4705920

[34] Cividini F , Scott BT , Dai A , et al. O‐GlcNAcylation of 8‐Oxoguanine DNA glycosylase (Ogg1) impairs oxidative mitochondrial DNA lesion repair in diabetic hearts. J Biol Chem. 2016;291(51):26515‐26528. 27816939 10.1074/jbc.M116.754481 PMC5159511

[35] Facundo HT , Brainard RE , Watson LJ , et al. O‐GlcNAc signaling is essential for NFAT‐mediated transcriptional reprogramming during cardiomyocyte hypertrophy. Am J Physiol Heart Circ Physiol. 2012;302:H2122‐H2130. 22408028 10.1152/ajpheart.00775.2011 PMC3362113

[36] Cannon MV , Sillje HH , Sijbesma JW , et al. Cardiac LXRα protects against pathological cardiac hypertrophy and dysfunction by enhancing glucose uptake and utilization. EMBO Mol Med. 2015;7(9):1229‐1243. 26160456 10.15252/emmm.201404669 PMC4568954

[37] Prakoso D , Lim SY , Erickson JR , et al. Fine‐tuning the cardiac O‐GlcNAcylation regulatory enzymes governs the functional and structural phenotype of the diabetic heart. Cardiovasc Res. 2022;118(1):212‐225. 33576380 10.1093/cvr/cvab043

[38] Kronlage M , Dewenter M , Grosso J , et al. O‐GlcNAcylation of histone deacetylase 4 protects the diabetic heart from failure. Circulation. 2019;140(7):580‐594. 31195810 10.1161/CIRCULATIONAHA.117.031942

[39] Hunter T . Protein kinases and phosphatases: the yin and yang of protein phosphorylation and signaling. Cell. 1995;80(2):225‐236. 7834742 10.1016/0092-8674(95)90405-0

[40] Hill MA , Yang Y , Zhang L , et al. Insulin resistance, cardiovascular stiffening and cardiovascular disease. Metabolism. 2021;119:154766. 33766485 10.1016/j.metabol.2021.154766

[41] Boucher J , Kleinridders A , Kahn CR . Insulin receptor signaling in normal and insulin‐resistant states. Cold Spring Harb Perspect Biol. 2014;6(1):a009191. 24384568 10.1101/cshperspect.a009191 PMC3941218

[42] Morisco C , Condorelli G , Trimarco V , et al. Akt mediates the cross‐talk between beta‐adrenergic and insulin receptors in neonatal cardiomyocytes. Circ Res. 2005;96(2):180‐188. 15591229 10.1161/01.RES.0000152968.71868.c3

[43] Marrero MB , Fulton D , Stepp D , Stern DM . Angiotensin II‐induced insulin resistance and protein tyrosine phosphatases. Arterioscler Thromb Vasc Biol. 2004;24(11):2009‐2013. 15271787 10.1161/01.ATV.0000140059.04717.f3

[44] Tabbi‐Anneni I , Buchanan J , Cooksey RC , Abel ED . Captopril normalizes insulin signaling and insulin‐regulated substrate metabolism in obese (ob/ob) mouse hearts. Endocrinology. 2008;149(8):4043‐4050. 18450963 10.1210/en.2007-1646 PMC2488224

[45] Ni YG , Wang N , Cao DJ , et al. FoxO transcription factors activate Akt and attenuate insulin signaling in heart by inhibiting protein phosphatases. Proc Natl Acad Sci U S A. 2007;104(51):20517‐20522. 18077353 10.1073/pnas.0610290104 PMC2154463

[46] Battiprolu PK , Hojayev B , Jiang N , et al. Metabolic stress‐induced activation of FoxO1 triggers diabetic cardiomyopathy in mice. J Clin Invest. 2012;122(3):1109‐1118. 22326951 10.1172/JCI60329 PMC3287230

[47] Huang J , Huang S , Deng J , Hung L . Impairment of insulin‐stimulated Akt/GLUT4 signaling is associated with cardiac contractile dysfunction and aggravates I/R injury in STZ‐diabetic rats. J Biomed Sci. 2009;16(1):77. 19706162 10.1186/1423-0127-16-77 PMC2740847

[48] Slot JW , Geuze HJ , Gigengack S , et al. Translocation of the glucose transporter GLUT4 in cardiac myocytes of the rat. Proc Natl Acad Sci U S A. 1991;88(17):7815‐7819. 1881917 10.1073/pnas.88.17.7815 PMC52394

[49] Szablewski L . Glucose transporters in healthy heart and in cardiac disease. Int J Cardiol. 2017;230:70‐75. 28034463 10.1016/j.ijcard.2016.12.083

[50] Hou N , Mai Y , Qiu X , et al. Carvacrol Attenuates Diabetic Cardiomyopathy by Modulating the PI3K/AKT/GLUT4 Pathway in Diabetic Mice. 10.3389/fphar.2019.00998 PMC6751321 31572181

[51] Liu J , Zhao Y , Zhu Y , et al. Rhynchophylline regulates calcium homeostasis by antagonizing ryanodine receptor 2 phosphorylation to improve diabetic cardiomyopathy. Front Pharmacol. 2022;13:882198. 35517784 10.3389/fphar.2022.882198 PMC9063879

[52] Hart GW , Slawson C , Ramirez‐Correa G , Lagerlof O . Cross talk between O‐GlcNAcylation and phosphorylation: roles in signaling, transcription, and chronic disease. Annu Rev Biochem. 2011;80:825‐858. 21391816 10.1146/annurev-biochem-060608-102511 PMC3294376

[53] Zhong X , Wang T , Zhang W , et al. ERK/RSK‐mediated phosphorylation of Y‐box binding protein‐1 aggravates diabetic cardiomyopathy by suppressing its interaction with deubiquitinase OTUB1. J Biol Chem. 2022;298(6):101989. 35490780 10.1016/j.jbc.2022.101989 PMC9163515

[54] Li Z , Xu S , Liu P . Salvia miltiorrhizaBurge (Danshen): a golden herbal medicine in cardiovascular therapeutics. Acta Pharmacol Sin. 2018;39(5):802‐824. 29698387 10.1038/aps.2017.193 PMC5943903

[55] Li C , Liu B , Wang Z , et al. Salvianolic acid B improves myocardial function in diabetic cardiomyopathy by suppressing IGFBP3. J Mol Cell Cardiol. 2020;139:98‐112. 31982427 10.1016/j.yjmcc.2020.01.009

[56] Hardie DG . Roles of protein kinases and phosphatases in signal transduction. Symp Soc Exp Biol. 1990;44:241‐255. 1966636

[57] Hartmann S , Ridley AJ , Lutz S . The function of rho‐associated kinases ROCK1 and ROCK2 in the pathogenesis of cardiovascular disease. Front Pharmacol. 2015;6:276. 26635606 10.3389/fphar.2015.00276 PMC4653301

[58] El‐Waseif AG , Nader MA , Salem HA , Elshaer SL . Fasudil, a ROCK inhibitor, preserves limb integrity in a mouse model of unilateral critical limb ischemia: possible interplay of inflammatory and angiogenic signaling pathways. Life Sci. 2022;309:121019. 36195296 10.1016/j.lfs.2022.121019

[59] Rocha S , Lucas M , Araújo AN , et al. Optimization and validation of an in vitro standardized glycogen phosphorylase activity assay. Molecules. 2021;26(15):4635. 34361792 10.3390/molecules26154635 PMC8347172

[60] Mattei AL , Bailly N , Meissner A . DNA methylation: a historical perspective. Trends Genet. 2022;38(7):676‐707. 35504755 10.1016/j.tig.2022.03.010

[61] Berulava T , Buchholz E , Elerdashvili V , et al. Changes in m6A RNA methylation contribute to heart failure progression by modulating translation. Eur J Heart Fail. 2020;22(1):54‐66. 31849158 10.1002/ejhf.1672

[62] Yi X , Zhu Q , Wu X , et al. Histone methylation and oxidative stress in cardiovascular diseases. Oxid Med Cell Longev. 2022;2022:6023710. 35340204 10.1155/2022/6023710 PMC8942669

[63] Carlson SM , Gozani O . Nonhistone lysine methylation in the regulation of cancer pathways. Cold Spring Harb Perspect Med. 2016;6(11):a026435. 27580749 10.1101/cshperspect.a026435 PMC5088510

[64] Shi Y , Zhang H , Huang S , et al. Epigenetic regulation in cardiovascular disease: mechanisms and advances in clinical trials. Signal Transduct Target Ther. 2022;7(1):200. 35752619 10.1038/s41392-022-01055-2 PMC9233709

[65] Xue W , Huang J , Chen H , et al. Histone methyltransferase G9a modulates hepatic insulin signaling via regulating HMGA1. Biochim Biophys Acta Mol Basis Dis. 2018;1864(2):338‐346. 29101051 10.1016/j.bbadis.2017.10.037

[66] El‐Osta A , Brasacchio D , Yao D , et al. Transient high glucose causes persistent epigenetic changes and altered gene expression during subsequent normoglycemia. J Exp Med. 2008;205(10):2409‐2417. 18809715 10.1084/jem.20081188 PMC2556800

[67] Brasacchio D , Okabe J , Tikellis C , et al. Hyperglycemia induces a dynamic cooperativity of histone methylase and demethylase enzymes associated with gene‐activating epigenetic marks that coexist on the lysine tail. Diabetes. 2009;58(5):1229‐1236. 19208907 10.2337/db08-1666 PMC2671038

[68] Gaikwad AB , Sayyed SG , Lichtnekert J , et al. Renal failure increases cardiac histone h3 acetylation, dimethylation, and phosphorylation and the induction of cardiomyopathy‐related genes in type 2 diabetes. Am J Pathol. 2010;176(3):1079‐1083. 20075197 10.2353/ajpath.2010.090528 PMC2832129

[69] Ou X , Zhu C , Sun S . Effects of obesity and diabetes on the epigenetic modification of mammalian gametes. J Cell Physiol. 2019;234(6):7847‐7855. 30536398 10.1002/jcp.27847

[70] Zhuang L , Jang Y , Park Y , et al. Depletion of Nsd2‐mediated histone H3K36 methylation impairs adipose tissue development and function. Nat Commun. 2018;9(1):1796. 29728617 10.1038/s41467-018-04127-6 PMC5935725

[71] Zheng Q , Cao Y , Chen Y , et al. Senp2 regulates adipose lipid storage by de‐SUMOylation of Setdb1. J Mol Cell Biol. 2018;10(3):258‐266. 29272473 10.1093/jmcb/mjx055

[72] Varma U , Koutsifeli P , Benson VL , et al. Molecular mechanisms of cardiac pathology in diabetes—experimental insights. Biochim Biophys Acta Mol Basis Dis. 2018;1864(5 Pt B):1949‐1959. 29109032 10.1016/j.bbadis.2017.10.035

[73] Yu X , Geng Y , Liang J , et al. High levels of glucose induce “metabolic memory” in cardiomyocyte via epigenetic histone H3 lysine 9 methylation. Mol Biol Rep. 2012;39(9):8891‐8898. 22707199 10.1007/s11033-012-1756-z

[74] Zhu N , Zhu L , Huang B , et al. Galectin‐3 inhibition ameliorates Streptozotocin‐induced diabetic cardiomyopathy in mice. Front Cardiovasc Med. 2022;9:868372. 35557520 10.3389/fcvm.2022.868372 PMC9086782

[75] Abukhalil MH , Althunibat OY , Aladaileh SH , et al. Galangin attenuates diabetic cardiomyopathy through modulating oxidative stress, inflammation and apoptosis in rats. Biomed Pharmacother. 2021;138:111410. 33752930 10.1016/j.biopha.2021.111410

[76] Hao J , Liu Y . Epigenetics of methylation modifications in diabetic cardiomyopathy. Front Endocrinol (Lausanne). 2023;14:1119765. 37008904 10.3389/fendo.2023.1119765 PMC10050754

[77] Yang M , Zhang Y , Ren J . Acetylation in cardiovascular diseases:Molecular Mmechanisms and Cclinical Iimplications. Biochim Biophys Acta Mol Basis Dis. 2020;1866(10):165836. 32413386 10.1016/j.bbadis.2020.165836

[78] Shvedunova M , Akhtar A . Modulation of cellular processes by histone and non‐histone protein acetylation. Nat Rev Mol Cell Biol. 2022;23(5):329‐349. 35042977 10.1038/s41580-021-00441-y

[79] Jankauskas SS , Kansakar U , Varzideh F , et al. Heart failure in diabetes. Metabolism. 2021;125:154910. 34627874 10.1016/j.metabol.2021.154910 PMC8941799

[80] Li L , Zeng H , He X , Chen J . Sirtuin 3 alleviates diabetic cardiomyopathy by regulating TIGAR and cardiomyocyte metabolism. J Am Heart Assoc. 2021;10(5):e018913. 33586458 10.1161/JAHA.120.018913 PMC8174281

[81] Alcendor RR , Gao S , Zhai P , et al. Sirt1 regulates aging and resistance to oxidative stress in the heart. Circ Res. 2007;100(10):1512‐1521. 17446436 10.1161/01.RES.0000267723.65696.4a

[82] Fernandes GFS , Silva GDB , Pavan AR , et al. Epigenetic regulatory mechanisms induced by resveratrol. Nutrients. 2017;9(11):1201. 29104258 10.3390/nu9111201 PMC5707673

[83] Guo R , Liu W , Liu B , et al. SIRT1 suppresses cardiomyocyte apoptosis in diabetic cardiomyopathy: an insight into endoplasmic reticulum stress response mechanism. Int J Cardiol. 2015;191:36‐45. 25965594 10.1016/j.ijcard.2015.04.245

[84] Bagul PK , Deepthi N , Sultana R , Banerjee SK . Resveratrol ameliorates cardiac oxidative stress in diabetes through deacetylation of NFkB‐p65 and histone 3. J Nutr Biochem. 2015;26(11):1298‐1307. 26298192 10.1016/j.jnutbio.2015.06.006

[85] Sulaiman M , Matta MJ , Sunderesan NR , et al. Resveratrol, an activator of SIRT1, upregulates sarcoplasmic calcium ATPase and improves cardiac function in diabetic cardiomyopathy. Am J Physiol Heart Circ Physiol. 2010;298(3):H833‐H843. 20008278 10.1152/ajpheart.00418.2009 PMC2838561

[86] Karbasforooshan H , Karimi G . The role of SIRT1 in diabetic cardiomyopathy. Biomed Pharmacother. 2017;90:386‐392. 28380414 10.1016/j.biopha.2017.03.056

[87] Bagul PK , Dinda AK , Banerjee SK . Effect of resveratrol on sirtuins expression and cardiac complications in diabetes. Biochem Biophys Res Commun. 2015;468(1–2):221‐227. 26518647 10.1016/j.bbrc.2015.10.126

[88] Hein S , Arnon E , Kostin S , et al. Progression from compensated hypertrophy to failure in the pressure‐overloaded human heart: structural deterioration and compensatory mechanisms. Circulation. 2003;107(7):984‐991. 12600911 10.1161/01.cir.0000051865.66123.b7

[89] Bugyei‐Twum A , Advani A , Advani SL , et al. High glucose induces Smad activation via the transcriptional coregulator p300 and contributes to cardiac fibrosis and hypertrophy. Cardiovasc Diabetol. 2014;13:89. 24886336 10.1186/1475-2840-13-89 PMC4108062

[90] Bodiga VL , Eda SR , Bodiga S . Advanced glycation end products: role in pathology of diabetic cardiomyopathy. Heart Fail Rev. 2014;19(1):49‐63. 23404649 10.1007/s10741-013-9374-y

[91] Cantó C , Gerhart‐Hines Z , Feige JN , et al. AMPK regulates energy expenditure by modulating NAD+ metabolism and SIRT1 activity. Nature. 2009;458(7241):1056‐1060. 19262508 10.1038/nature07813 PMC3616311

[92] Hubbard BP , Sinclair DA . Small molecule SIRT1 activators for the treatment of aging and age‐related diseases. Trends Pharmacol Sci. 2014;35(3):146‐154. 24439680 10.1016/j.tips.2013.12.004 PMC3970218

[93] Chen Y , Du J , Zhao YT , et al. Histone deacetylase (HDAC) inhibition improves myocardial function and prevents cardiac remodeling in diabetic mice. Cardiovasc Diabetol. 2015;14:99. 26245924 10.1186/s12933-015-0262-8 PMC4527099

[94] Berthiaume JM , Kurdys JG , Muntean DM , Rosca MG . Mitochondrial NAD+/NADH redox state and diabetic cardiomyopathy. Antioxid Redox Signal. 2019;30(3):375‐398. 29073779 10.1089/ars.2017.7415 PMC6306679

[95] Tong D , Schiattarella GG , Jiang N , et al. NAD+ repletion reverses heart failure with preserved ejection fraction. Circ Res. 2021;128(11):1629‐1641. 33882692 10.1161/CIRCRESAHA.120.317046 PMC8159891

[96] Paramesha B , Anwar MS , Meghwani H , et al. Sirt1 and Sirt3 activation improved cardiac function of diabetic rats via modulation of mitochondrial function. Antioxidants (Basel). 2021;10(3):338. 33668369 10.3390/antiox10030338 PMC7996143

[97] Jin L , Geng L , Ying L , et al. FGF21‐Sirtuin 3 axis confers the protective effects of exercise against diabetic cardiomyopathy by governing mitochondrial integrity. Circulation. 2022;146(20):1537‐1557. 36134579 10.1161/CIRCULATIONAHA.122.059631

[98] Varshavsky A . The ubiquitin system, autophagy, and regulated protein degradation. Annu Rev Biochem. 2017;86:123‐128. 28654326 10.1146/annurev-biochem-061516-044859

[99] Smalle J , Vierstra RD . The ubiquitin 26S proteasome proteolytic pathway. Annu Rev Plant Biol. 2004;55:555‐590. 15377232 10.1146/annurev.arplant.55.031903.141801

[100] Zheng N , Shabek N . Ubiquitin ligases: structure, function, and regulation. Annu Rev Biochem. 2017;86:129‐157. 28375744 10.1146/annurev-biochem-060815-014922

[101] Toma‐Fukai S , Shimizu T . Structural diversity of ubiquitin E3 ligase. Molecules. 2021;26(21):6682. 34771091 10.3390/molecules26216682 PMC8586995

[102] Yang X , Xiang D , Yang Y . Role of E3 ubiquitin ligases in insulin resistance. Diabetes Obes Metab. 2016;18(8):747‐754. 27097743 10.1111/dom.12677

[103] Shi J , Luo L , Eash J , et al. The SCF‐Fbxo40 complex induces IRS1 ubiquitination in skeletal muscle, limiting IGF1 signaling. Dev Cell. 2011;21(5):835‐847. 22033112 10.1016/j.devcel.2011.09.011

[104] Yi J , Park JS , Ham Y , et al. MG53‐induced IRS‐1 ubiquitination negatively regulates skeletal myogenesis and insulin signalling. Nat Commun. 2013;4:2354. 23965929 10.1038/ncomms3354 PMC3941707

[105] Scheufele F , Wolf B , Kruse M , et al. Evidence for a regulatory role of Cullin‐RING E3 ubiquitin ligase 7 in insulin signaling. Cell Signal. 2014;26(2):233‐239. 24219910 10.1016/j.cellsig.2013.11.005 PMC3901049

[106] Sarikas A , Xu X , Field LJ , Pan Z . The cullin7 E3 ubiquitin ligase: a novel player in growth control. Cell Cycle. 2008;7(20):3154‐3161. 18927510 10.4161/cc.7.20.6922 PMC2637179

[107] Tsushima K , Bugger H , Wende AR , et al. Mitochondrial reactive oxygen species in Lipotoxic hearts induce post‐translational modifications of AKAP121, DRP1, and OPA1 that promote mitochondrial fission. Circ Res. 2018;122(1):58‐73. 29092894 10.1161/CIRCRESAHA.117.311307 PMC5756120

[108] Merrill RA , Strack S . Mitochondria: a kinase anchoring protein 1, a signaling platform for mitochondrial form and function. Int J Biochem Cell Biol. 2014;48:92‐96. 24412345 10.1016/j.biocel.2013.12.012 PMC3940257

[109] Schmitt K , Grimm A , Dallmann R , et al. Circadian control of DRP1 activity regulates mitochondrial dynamics and bioenergetics. Cell Metab. 2018;27(3):657‐666. 29478834 10.1016/j.cmet.2018.01.011

[110] Frezza C , Cipolat S , Brito OM , et al. OPA1 controls apoptotic cristae remodeling independently from mitochondrial fusion. Cell. 2006;126(1):177‐189. 16839885 10.1016/j.cell.2006.06.025

[111] Geng F , Wenzel S , Tansey WP . Ubiquitin and proteasomes in transcription. Annu Rev Biochem. 2012;81:177‐201. 22404630 10.1146/annurev-biochem-052110-120012 PMC3637986

[112] Rodríguez JE , Liao J , He J , et al. The ubiquitin ligase MuRF1 regulates PPARα activity in the heart by enhancing nuclear export via monoubiquitination. Mol Cell Endocrinol. 2015;413:36‐48. 26116825 10.1016/j.mce.2015.06.008 PMC4523404

[113] Wang Y , Sun W , Du B , et al. Therapeutic effect of MG‐132 on diabetic cardiomyopathy is associated with its suppression of proteasomal activities: roles of Nrf2 and NF‐κB. Am J Physiol Heart Circ Physiol. 2013;304(4):H567‐H578. 23220333 10.1152/ajpheart.00650.2012

[114] Finck BN , Lehman JJ , Leone TC , et al. The cardiac phenotype induced by PPARalpha overexpression mimics that caused by diabetes mellitus. J Clin Invest. 2002;109(1):121‐130. 11781357 10.1172/JCI14080 PMC150824

[115] Gomes MD , Lecker SH , Jagoe RT , et al. Atrogin‐1, a muscle‐specific F‐box protein highly expressed during muscle atrophy. Proc Natl Acad Sci U S A. 2001;98(25):14440‐14445. 11717410 10.1073/pnas.251541198 PMC64700

[116] Li H , Kedar V , Zhang C , et al. Atrogin‐1/muscle atrophy F‐box inhibits calcineurin‐dependent cardiac hypertrophy by participating in an SCF ubiquitin ligase complex. J Clin Invest. 2004;114(8):1058‐1071. 15489953 10.1172/JCI22220 PMC522252

[117] Ye B , Zhou H , Chen Y , et al. USP25 ameliorates pathological cardiac hypertrophy by stabilizing SERCA2a in cardiomyocytes. Circ Res. 2023;132(4):465‐480. 36722348 10.1161/CIRCRESAHA.122.321849

[118] Chen H , Moreno‐Moral A , Pesce F , et al. WWP2 regulates pathological cardiac fibrosis by modulating SMAD2 signaling. Nat Commun. 2019;10(1):3616. 31399586 10.1038/s41467-019-11551-9 PMC6689010

[119] Adams V , Schauer A , Augstein A , et al. Targeting MuRF1 by small molecules in a HFpEF rat model improves myocardial diastolic function and skeletal muscle contractility. J Cachexia Sarcopenia Muscle. 2022;13(3):1565‐1581. 35301823 10.1002/jcsm.12968 PMC9178400

[120] Riehle C , Abel ED . Insulin signaling and heart failure. Circ Res. 2016;118(7):1151‐1169. 27034277 10.1161/CIRCRESAHA.116.306206 PMC4833475

[121] Kashiwagi A , Obata T , Suzaki M , et al. Increase in cardiac muscle fructose content in streptozotocin‐induced diabetic rats. Metabolism. 1992;41(10):1041‐1046. 1406291 10.1016/0026-0495(92)90283-g

[122] Lal S , Randall WC , Taylor AH , et al. Fructose‐3‐phosphate production and polyol pathway metabolism in diabetic rat hearts. Metabolism. 1997;46(11):1333‐1338. 9361695 10.1016/s0026-0495(97)90240-7

[123] Delbridge LMD , Benson VL , Ritchie RH , Mellor KM . Diabetic cardiomyopathy: the case for a role of fructose in disease etiology. Diabetes. 2016;65(12):3521‐3528. 27879401 10.2337/db16-0682

[124] Alves‐Figueiredo H , Silva‐Platas C , Lozano O , et al. A systematic review of post‐translational modifications in the mitochondrial permeability transition pore complex associated with cardiac diseases. Biochim Biophys Acta Mol Basis Dis. 2021;1867(1):165992. 33091565 10.1016/j.bbadis.2020.165992

[125] Page LML , Rider OJ , Lewis AJ , et al. Increasing pyruvate dehydrogenase flux as a treatment for diabetic cardiomyopathy: a combined 13C hyperpolarized magnetic resonance and echocardiography study. Diabetes. 2015;64(8):2735‐2743. 25795215 10.2337/db14-1560 PMC4516266

[126] Shi Y , Jia C , Xu W , et al. Chidamide in relapsed or refractory peripheral T cell lymphoma: a multicenter real‐world study in China. J Hematol Oncol. 2017;10(1):69. 28298231 10.1186/s13045-017-0439-6 PMC5351273

[127] Sharma S , Taliyan R . Histone deacetylase inhibitors: future therapeutics for insulin resistance and type 2 diabetes. Pharmacol Res. 2016;113(Pt A):320‐326. 27620069 10.1016/j.phrs.2016.09.009

[128] Greenstein JL , Winslow RL . Integrative systems models of cardiac excitation‐contraction coupling. Circ Res. 2011;108(1):70‐84. 21212390 10.1161/CIRCRESAHA.110.223578 PMC3074965

[129] Györke S , Carnes C . Dysregulated sarcoplasmic reticulum calcium release: potential pharmacological target in cardiac disease. Pharmacol Ther. 2008;119(3):340‐354. 18675300 10.1016/j.pharmthera.2008.06.002 PMC2798594

[130] Zhihao L , Jingyu N , Lan L , et al. SERCA2a: a key protein in the Ca2+ cycle of the heart failure. Heart Fail Rev. 2020;25(3):523‐535. 31701344 10.1007/s10741-019-09873-3

[131] Park HJ , Choi EK , Choi J , et al. Heat‐induced up‐regulation of NAD(P)H:quinone oxidoreductase potentiates anticancer effects of beta‐lapachone. Clin Cancer Res. 2005;11(24 Pt 1):8866‐8871. 16361576 10.1158/1078-0432.CCR-05-0818

[132] Gorski PA , Jang SP , Jeong D , et al. Role of SIRT1 in modulating acetylation of the Sarco‐endoplasmic reticulum Ca2+‐ATPase in heart failure. Circ Res. 2019;124(9):e63‐e80. 30786847 10.1161/CIRCRESAHA.118.313865 PMC6483854

[133] Tian C , Zhang J , Liu J , et al. Ryanodine receptor and immune‐related molecules in diabetic cardiomyopathy. ESC Heart Fail. 2021;8(4):2637‐2646. 34013670 10.1002/ehf2.13431 PMC8318495

[134] Gambardella J , Jankauskas SS , D'Ascia SL , et al. Glycation of ryanodine receptor in circulating lymphocytes predicts the response to cardiac resynchronization therapy. J Heart Lung Transplant. 2022;41(4):438‐441. 35042640 10.1016/j.healun.2021.12.008 PMC8977242

